# Endocardial Ablation of Atrial Flutter with Involvement of the Vein of Marshall: A Case Report

**DOI:** 10.3390/jcm14134598

**Published:** 2025-06-29

**Authors:** Lucio Addeo, Konstantinos Triantafyllou, Hellen Dockx, Monika Shumkova, Antonio Rapacciuolo, Stefano Nardi, Vittoria Marino, Luigi Argenziano, Pasquale Guarini, Laura Adelaide Dalla Vecchia, Francesco Donatelli, Tom De Potter

**Affiliations:** 1 Cardiovascular Center Aalst, Azorg Hospital, 9300 Aalst, Belgium; 2 Department of Advanced Biomedical Sciences, University of Naples Federico II, 80138 Naples, Italy; 3 Pineta Grande Hospital, 81030 Castel Volturno, Italy; 4 U.O Cardiologia, Clinica Sanatrix, Centro Studi SICOA, 80127 Naples, Italy; 5 Department of Cardiology, IRCCS Istituti Clinici Scientifici Maugeri, 20138 Milan, Italy; 6 Department of Clinical and Community Sciences, University of Milan, 20122 Milan, Italy

**Keywords:** atrial flutter, epicardial circuit, Vein of Marshall, catheter ablation, case report

## Abstract

**Background:** Atypical atrial flutter (AFL) is a complex clinical challenge, particularly in patients with prior atrial fibrillation (AF) treated with pulmonary vein isolation (PVI). Arrhythmias involving the vein of Marshall (VOM) often require extensive lesion sets, including ethanol infusion, to effectively target the epicardial substrate. To minimize tissue damage, an alternative strategy has been proposed, emphasizing advanced electroanatomical mapping, entrainment maneuvers, and highly targeted ablation techniques. **Case Presentation:** We describe a 72-year-old woman with recurrent atrial arrhythmias following pulmonary vein isolation (PVI), who presented with palpitations as her primary symptom. After ineffective pharmacological therapy, she underwent a catheter ablation procedure. Electroanatomical mapping revealed significant left atrial scarring and suggested a macroreentrant circuit involving the VOM. Entrainment maneuvers confirmed the VOM’s involvement. A single targeted endocardial ablation guided by the ablation index terminated the arrhythmia within 12 s, without the need for ethanol infusion or extensive lesion sets. **Discussion:** This case underscores the VOM’s role in sustaining atypical AFL post-PVI and highlights the effectiveness of precise electroanatomical mapping combined with targeted endocardial ablation. Unlike broader ablation or ethanol infusion strategies, a focused lesion at the critical isthmus achieved arrhythmia termination with minimal tissue damage. **Conclusions:** Endocardial ablation at the site of entrainment can safely and effectively treat VOM-related AFL, offering symptom relief and restoration of sinus rhythm. This approach may reduce procedural risks and expand the feasibility of VOM-related arrhythmia management in centers without access to ethanol infusion.

## 1. Introduction

Atrial arrhythmias remain a significant clinical challenge, especially in patients with a history of atrial fibrillation (AF) previously treated with catheter ablation. Atypical atrial flutter (AFL) can represent a complex form of recurrence, often requiring advanced diagnostic and therapeutic approaches. This report highlights the successful management of an atypical atrial flutter with a critical isthmus involving an epicardial component (the vein of Marshall—VOM), emphasizing the importance of detailed electroanatomical mapping and targeted ablation. It also demonstrates the feasibility of a safe and effective endocardial approach in such cases.

## 2. Case Presentation

**Figure 1:**
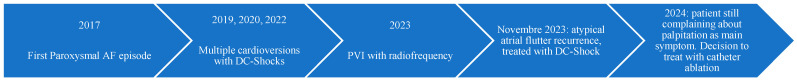
Timeline with relevant data from the episode of care.

The patient was a 72-year-old woman with a history of dyslipidemia as a cardiovascular risk factor. She experienced her first episode of paroxysmal AF in 2017, which was followed by recurrent episodes requiring multiple direct-current (DC) cardioversions in 2019, 2020, and 2022. In February 2023, she underwent pulmonary vein isolation (PVI) with radiofrequency (RF) ablation. However, AFL recurred in November 2023, necessitating another DC cardioversion (Figure 1). In 2024, persistent atypical AFL was documented on a surface electrocardiogram (ECG) and the patient was still complaining about palpitations as the main symptom.

**Figure 2:**
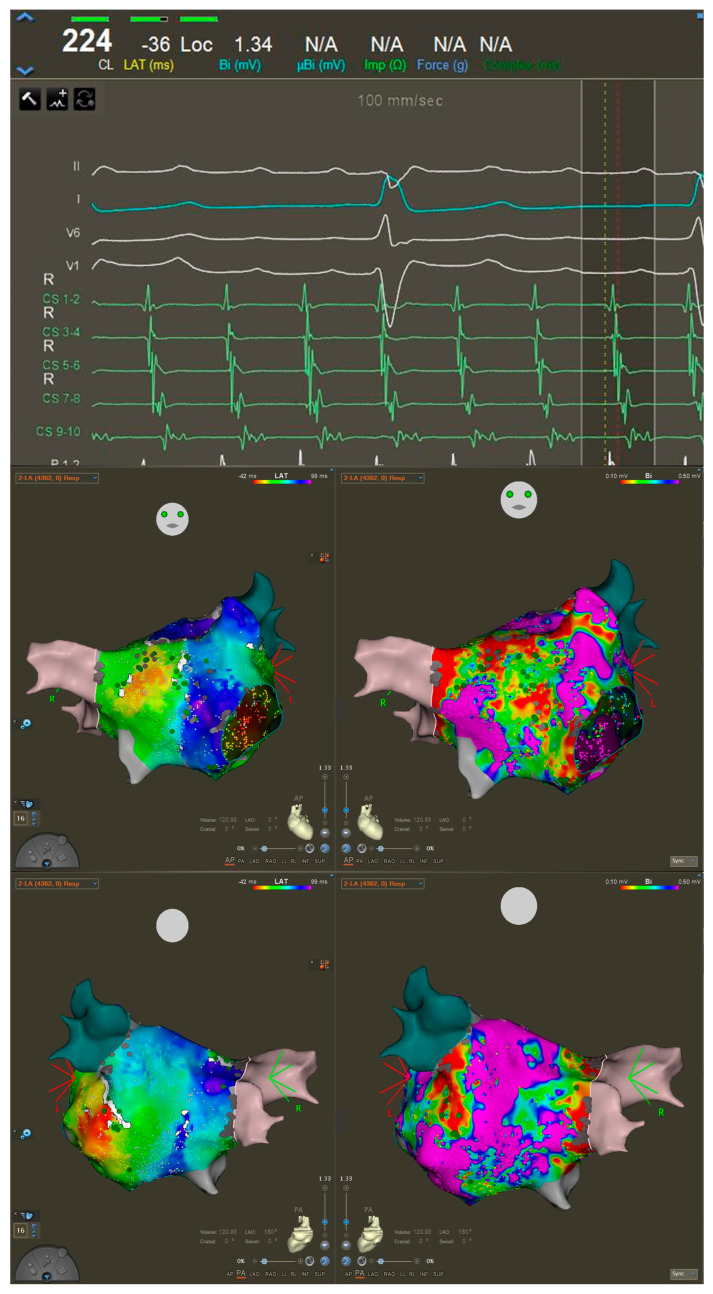
Activation pattern through the CS of the atypical AFL. Below, bipolar and LAT maps of anterior and posterior wall of LA.

**Figure 3:**
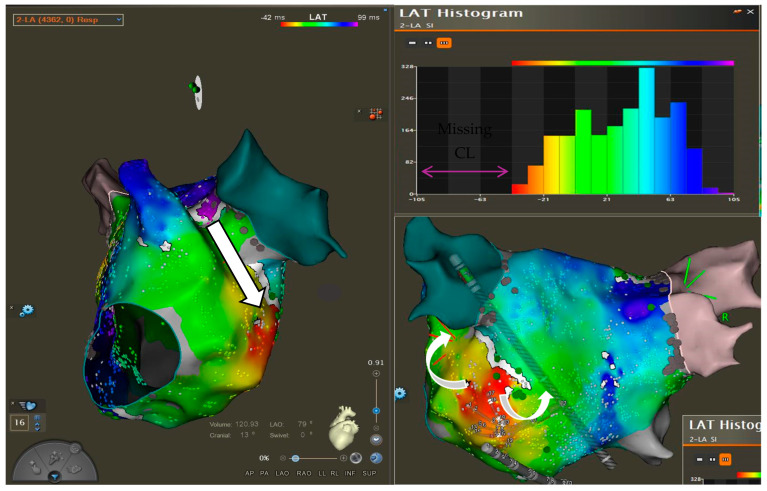
LAT Histogram, showing missing part of the CL. Analysis of the earliest and latest activation points led to an accurate diagnosis of the tachycardia mechanism.

**Figure 4:**
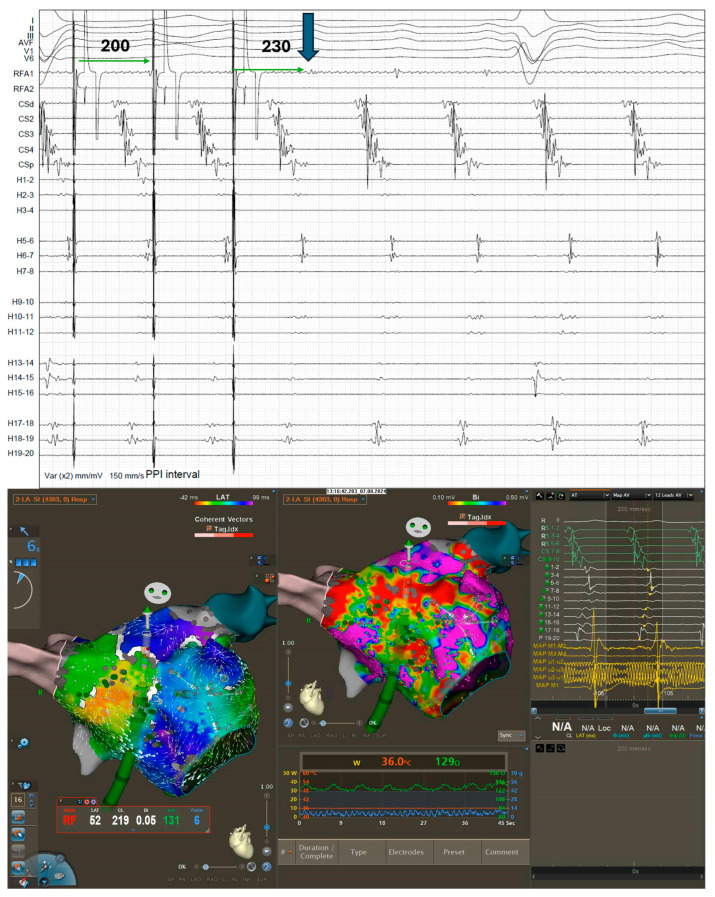
Entrainment maneuver from the base of the appendage showed a post-pacing interval of 230 ms, consistent with the CL of the tachycardia. In the same site, the ablation has been performed with successful restoration of sinus rhythm.

The patient was briefly treated with Sotalol (80 mg twice per day) without success, leaving catheter ablation as the only viable option for symptom control. Laboratory testing and echocardiographic data did not raise any relevant issue and showed a preserved ejection fraction. At the time of the procedure, she was on a regimen of Xarelto (20 mg/day) but was not taking any antiarrhythmic medications. The day of the procedure, the 12-lead surface ECG showed the same stable arrhythmia. The procedure was performed under general anesthesia with continuous monitoring of vital parameters. During the electrophysiological procedure, a stable AFL with a cycle length (CL) of 230 ms was documented. The standard mapping workflow included the use of a coronary sinus (CS) catheter, a PentaRay mapping catheter, and a Qdot ablation catheter. After the insertion of the CS catheter, signals demonstrated a distal-to-proximal activation pattern, consistent with a flutter originating from the left atrium (Figure 2). A double transseptal puncture was performed to access the left atrium. The anatomy of the chamber was reconstructed by electroanatomical 3D mapping, using the CARTO system (Biosense Webster, Irvine, CA, USA). Both bipolar and local activation time (LAT) maps, revealed significant areas of scar on the anterior wall of the left atrium, whereas the posterior wall displayed relatively preserved tissue integrity (Figure 1). The local-activation-time (LAT) histogram (Figure 2) plots how long each mapped point of atrial tissue takes to activate during one full cycle of the tachycardia. Ideally, if every part of the circuit activates in sequence (without any “missing” time), the bars would form a continuous band that fills the entire CL. In our case, a noticeable empty segment appeared in the histogram. At first glance, that gap suggested a focal atrial tachycardia: if a single spot on the posterior wall were firing automatically, the wavefront would spread outward, and many mapping points would activate almost simultaneously, leaving an unused portion of the CL in which no new tissue is being excited. Considering LAT values in the context of the three-dimensional electro-anatomical map, we noticed that the earliest and latest activation points were right next to each other in space, effectively “closing the loop.” In addition, the activation sequence wrapped around an area that our endocardial catheter could not directly record. This pattern is much more consistent with a macro-reentrant circuit that slips into or through tissue on the epicardial surface, working as bypass, that the catheter cannot see, rather than with a true focal source. The spatial evidence therefore clarified that the tachycardia most likely travels along an epicardial route (Figure 3) even though the LAT histogram alone first pointed us toward a focal mechanism and that the mechanism of the arrhythmia was hypothesized to involve the VOM, based on the earliest activation zones and observed propagation patterns. Entrainment maneuvers performed at a CL of 200 ms from the base of the left atrial appendage, in front of the left superior pulmonary vein, confirmed the VOM’s involvement in the flutter circuit, as demonstrated by a post-pacing interval of 230 ms, consistent with the tachycardia CL (Figure 3). A single ablation, guided by the ablation index at the site of entrainment (Figure 4), was performed using the Qdot catheter (Biosense Webster) at 50 W. The tachycardia terminated after 12 s. We continued the application until we achieved an ablation index of 550 (assessing a contemporary impedance drop of at least 10% from the baseline). The site of application was reinforced with two additional ablations at adjacent locations, following the same criteria. Subsequently, all attempts to reinduce the flutter using programmed and decremental stimulation from the proximal CS failed to provoke the tachycardia, and the veins were still isolated. The procedural time was 67 min. At the 1-month follow-up, the patient remained in sinus rhythm without the need for antiarrhythmic medication and reported no symptoms with a relevant relief.

## 3. Discussion

This case underscores the challenge of managing AFL in a patient with a history of AF and prior point-by-point radiofrequency PVI. Although durable isolation of the pulmonary veins was confirmed, the subsequent appearance of atypical AFL highlights the risk of post-ablation arrhythmias and the need to understand their underlying mechanisms. In our view, the interplay between the earlier ablation and progressive left-atrial disease fostered the maintenance of this complex arrhythmia. The findings from electroanatomical mapping in this patient suggest that the AFL was driven by a macroreentrant circuit involving the VOM. The earliest activation points and propagation patterns identified during mapping were critical in formulating this hypothesis. Entrainment maneuvers confirmed the VOM’s role in sustaining the arrhythmia, with a post-pacing interval matching the tachycardia CL. This aligns with existing literature that implicates the VOM in atypical left atrial arrhythmias, particularly in cases where prior ablations may alter the electrophysiological substrate. Three percutaneous strategies can be used to interrupt a macro-reentrant circuit involving the vein of Marshall: an epicardial approach that accesses the pericardial space in a pericardiocentesis-like fashion, an endocardial approach in which ethanol is infused into the vein of Marshall via the coronary sinus, and an endocardial approach that ablates the circuit with standard catheters; these approaches can also be combined to achieve a more definitive lesion set when necessary.

Several case reports and series have documented the VOM as a key arrhythmogenic substrate in similar contexts. For instance, previous studies already reported the VOM as one of the main substrates after PVI [1]. Kitamura et al. [2] successively reported the successful use of selective ethanol ablation of the distal VOM to terminate a Marshall bundle–related atrial tachycardia, emphasizing the importance of precise targeting within the VOM. Similarly, Valderrabano et al. [3,4] highlighted the role of VOM ethanol infusion as an adjunctive therapy for AF treatment, noting its potential to address myocardial connections and arrhythmogenic foci. Furthermore, a previous study [5] described a left atrial roof-dependent tachycardia involving the VOM, underscoring the complexity of arrhythmias associated with this structure. It is important to note that ethanol infusion of the vein of Marshall, particularly in the context of prior ablation, may create an extensive area of scarring, potentially increasing the risk of new arrhythmias in the future. In contrast, ablation at the site of entrainment in this case successfully terminated the arrhythmia within 12 s, and the additional applications provided robust modification of the arrhythmogenic substrate without the need to ablate a broader region. Ablation was guided by the ablation index, with a target value of 550, while monitoring the impedance drop; in our case, impedance decreased by about 10% from the start of the lesion. This targeted ablation strategy highlights the importance of precise mapping and the utility of the ablation index in achieving durable lesion formation, demonstrating that endocardial ablation is feasible, also in circuits with both epicardial and endocardial routes. The total procedure time was 67 min, indicating that this approach does not necessarily prolong the intervention; conversely, this strategy may even reduce procedure time compared with VOM ablation using ethanol infusion. [6]. Despite acute success, a key limitation of the endocardial-only approach is that it may fail to produce a fully transmural lesion, a result that can vary with patient-related factors such as atrial-wall thickness (e.g., hypertrophy) or the presence of extensive fibrosis. The follow-up visit revealed a significant clinical improvement, especially regarding the symptoms. The failure of pharmacological therapy in this patient underscores the limitations of antiarrhythmic drugs in managing these complex and stable arrhythmias and highlights catheter ablation as a valuable treatment option even in this setting providing symptomatic relief and restoring sinus rhythm in similar cases. While this case demonstrates a successful outcome, the follow-up period is relatively short, and longer-term monitoring is needed to confirm the durability of the ablation and the maintenance of sinus rhythm. Finally, the emergence of new arrhythmias originating near existing or procedure-related scars cannot be excluded and should always be considered during subsequent evaluations. Innovative, non-invasive imaging, particularly speckle-tracking echocardiography (STE), can help identify patients at higher risk of arrhythmia recurrence. Left-atrial strain measured with STE predicts relapse after catheter ablation [7] and after electrical cardioversion [8]. Integrating STE into routine pre- and post-procedural echocardiographic assessments could therefore guide more tailored follow-up strategies.

## 4. Conclusions

This case emphasizes the importance of detailed electroanatomical mapping and precise entrainment maneuvers in identifying atypical flutter circuits involving epicardial structures such as the VOM. It demonstrates that a carefully targeted endocardial ablation can effectively and safely terminate such arrhythmias, avoiding the need for more extensive interventions like ethanol infusion. This approach may reduce procedural risks and expand the feasibility of VOM-related arrhythmia management in centers without access to ethanol infusion. Catheter ablation remains a crucial therapeutic option for patients with complex, drug-refractory atrial arrhythmias, offering significant symptomatic relief and restoration of sinus rhythm.

## Data Availability

The original contributions presented in this study are included in the article. Further inquiries can be directed to the corresponding author (addeolucio@gmail.com).

## Abbreviations

The following abbreviations are used in this manuscript:

AF	Atrial Fibrillation
AFL	Atrial Flutter
CL	Cycle Length
CS	Coronary Sinus
DC	Direct Current
ECG	Electrocardiogram
LAT	Local Activation Time
PVI	Pulmonary Vein Isolation
RF	Radiofrequency
STE	Speckle-tracking Echocardiography
VOM	Vein Of Marshall

## References

[1] Vlachos K., Denis A., Takigawa M., Kitamura T., Martin C.A., Frontera A., Martin R., Bazoukis G., Bourier F., Cheniti G. (2019). The role of Marshall bundle epicardial connection in atrial tachycardias after atrial fibrillation ablation. Heart Rhythm.

[2] Kitamura T., Vlachos K., Denis A., Andre C., Martin R., Pambrun T., Duchateau J., Frontera A., Takigawa M., Thompson N. (2019). Ethanol infusion for Marshall bundle epicardial connections in Marshall bundle-related atrial tachycardias following atrial fibrillation ablation: The accessibility and success rate of ethanol infusion by using a femoral approach. J. Cardiovasc. Electrophysiol..

[3] Valderrabano M. (2021). Vein of Marshall Ethanol Infusion in the Treatment of Atrial Fibrillation: From Concept to Clinical Practice. Heart Rhythm.

[4] Valderrábano M., Peterson L.E., Swarup V., Schurmann P.A., Makkar A., Doshi R.N., DeLurgio D., Athill C.A., Ellenbogen K.A., Natale A. (2020). Effect of Catheter Ablation With Vein of Marshall Ethanol Infusion vs Catheter Ablation Alone on Persistent Atrial Fibrillation The VENUS Randomized Clinical Trial. J. Am. Med. Assoc..

[5] Sasaki W., Nakamura K., Minami K., Sasaki T., Take Y., Naito S. (2021). Left atrial roof-dependent atrial tachycardia via the Marshall bundle. Heart Rhythm.

[6] Pambrun T., Denis A., Duchateau J., Sacher F., Hocini M., Jaïs P., Haïssaguerre M., Derval N. (2019). MARSHALL bundles elimination, Pulmonary veins isolation and Lines completion for ANatomical ablation of persistent atrial fibrillation: MARSHALL-PLAN case series. J. Cardiovasc. Electrophysiol..

[7] Motoc A., Luchian M.L., Scheirlynck E., Roosens B., Chameleva H., Gevers M., Galloo X., von Kemp B., Ramak R., Sieira J. (2021). Incremental value of left atrial strain to predict atrial fibrillation recurrence after cryoballoon ablation. PLoS ONE.

[8] Vincenti A., Genovesi S., Sonaglioni A., Binda G., Rigamonti E., Lombardo M., Anza C. (2019). Mechanical atrial recovery after cardioversion in persistent atrial fibrillation evaluated by bidimensional speckle tracking echocardiography. J. Cardiovasc. Med..

